# Introducing a Step-by-Step Guide to ARCP and Portfolio for Higher Trainees in KMPT

**DOI:** 10.1192/bjo.2025.10290

**Published:** 2025-06-20

**Authors:** Maria Moisan

**Affiliations:** Kent and Medway NHS and Social Care Partnership Trust, Maidstone, United Kingdom

## Abstract

**Aims:** This presentation aims to support psychiatry trainees, especially those new to the UK system or returning after a break, in navigating the complexities of the new curriculum and its requirements. The introduction of the Placement-Specific Personal Development Plan (PSPDP), Higher Level Outcomes (HLOs), has created a more structured but demanding framework. This guide helps trainees understand and manage these requirements, offering a condensed and practical overview of the portfolio and ARCP process. By focusing on resilience and capability, this presentation simplifies the guidelines provided by the Royal College of Psychiatrists (RCPsych), including the Silver Guide.

**Methods:** The presentation uses practical strategies and case reports to highlight the common challenges faced by higher trainees. Key issues include managing an active portfolio, mapping activities to HLOs and competencies, and fulfilling WPBA requirements. Real-life examples provide insights on how to set up and maintain portfolios, assign supervisors, and plan development in line with the new curriculum. The content offers practical solutions for trainees, particularly those new to the system or returning after a break. It is also valuable for clinical and educational supervisors, training programme directors (TPDs), and postgraduate medical education (PGME) staff who support trainees’ progression.

**Results:** The presentation was well-received in local teaching sessions, with trainees appreciating the clarity and structure it provided for understanding the new curriculum. Feedback suggested the practical guidance and step-by-step approach helped trainees feel more confident in managing portfolios and meeting new requirements. The discussion focused on engaging with the new system, the documentation processes, and balancing clinical duties with meeting competencies. Early planning, clear communication with supervisors, and a methodical approach to organizing the portfolio were emphasized to ensure the successful completion of assessments and documentation. This session, designed from the trainee’s perspective, has also been beneficial for supervisors and educators in understanding the challenges faced by trainees.

**Conclusion:** This presentation supports trainees, especially those unfamiliar with the UK system or returning after a gap, in navigating the complexities of the new curriculum. Feedback indicates the presentation successfully demystified the process and highlighted the resilience needed to meet the challenges. It will be included in the KSS Higher Trainees Induction and can become a regular teaching slot to provide ongoing support. A survey will be developed to gather formal feedback, improving the presentation for future trainees. The condensed content, based on RCPsych materials, makes extensive resources more accessible for trainees and their supervisors.

